# Ten simple rules for serving as an editor

**DOI:** 10.1371/journal.pcbi.1010911

**Published:** 2023-03-02

**Authors:** Cameron Mura, Philip E. Bourne

**Affiliations:** 1 School of Data Science, University of Virginia, Charlottesville, Virginia, United States of America; 2 Department of Biomedical Engineering, University of Virginia, Charlottesville, Virginia, United States of America; Carnegie Mellon University, UNITED STATES

This is a *PLOS Computational Biology* Methods paper.

## Introduction

We believe that readers, particularly those at relatively early career stages, could benefit from ten simple rules (TSR) on the topic of “serving as an editor.” By this phrase, we mean that role which may variously be called “handling editor,” “academic editor,” “scientific editor” and so on—in other words, the individual who oversees the process of shepherding a written piece of scientific work from the point of manuscript submission through to peer review and, ultimately, either publication or rejection. We mean this in contradistinction to, say, serving as an editorial advisory board member, a “section editor,” or an editor-in-chief or such. Those roles are of interest too, in terms of career and professional development; however, those types of positions generally begin later in one’s career (e.g., as a well-established scientist), versus nearer the start of an independent career (e.g., late-postdoc or early-faculty), spurring us to focus this piece more on the context of “handling editor.”

The goal of this TSR is to offer guidance that can help you, the reader—in your current or future roles as a novice handling editor—be the type of editor whom you might have liked to have dealt with yourself, in your own experience and interactions thus far in publishing your work. This piece can be viewed as complementary to an early TSR for reviewers [1], and the closest material of which we are aware is Erren and Erren’s “*Simple Rules for Editors*’? *Here is One Rule to Tackle Neglected Problems of Publishing*,” published as a correspondence in this journal 15 years ago [2]. That insightful piece suggested an “Editor Rule for Appropriate Recognition” as a way to improve the recognition and credit due to those who contribute to a submitted work, and yet who may be less visible or even entirely overlooked (possibly to the point of omission from an author list), perhaps because they are relatively young or less experienced than more senior authors. Specifically, Erren and Erren’s proposed “rule” called on editors to ask authors for a written statement that (i) avows that no substantial contributors have been omitted from an authorship list; and (ii) explicitly delineates the oftentimes key role of more junior/overlooked authors (and/or researchers that the work cites) in formulating the hypotheses or rationale that underlies the submitted study. We agree with those proposed practices. In the present work, we focus chiefly on the mechanics and best practices of one’s editorial responsibilities when handling a manuscript, starting at the initial point of being invited to serve as an editor.

## Rule 1: Only accept an invitation if you can do it—be dedicated to it, and ensure you can realistically prioritize it

Implied here is the notion that this is a role in the service of others and not yourself. Therefore, with self-preservation in mind, your undertaking this activity should be weighed seriously relative to your career stage, your long-term career objectives, and so on. Serving as an editor will take time and effort, and you should be able and willing to commit the necessary time and focus going into it; otherwise, don’t do it, lest it be a disservice to all involved. (While it may feel like a missed opportunity, this type of opportunity/invitation will surely arise again.) As with all potential obligations, don’t commit to an editorial one if you do not have sufficient time or “bandwidth.” This is essentially the “learn to say *No*” principle.

An addendum to this Rule is to accept an editorial invitation if you believe it makes sense to, in terms of scientific overlap with your own background and expertise. For example, is this a journal that you would typically publish in yourself? If not, perhaps you should not be an editor for it? This is an important point not only in terms of scientific overlap, but also because of a secondary effect that relates to human nature: The more “involved” you are with a particular journal (e.g., as someone who publishes in it), the more likely you are to be invested in a high-quality editorial process.

## Rule 2: Draw upon your experiences to guide your decision-making as an editor—*Look backward* (on your own experiences), and *look forward* (with appreciation and respect)

Looking backward, use your own history of publishing and authorship efforts as a guide. Reflect on and study the possibly many examples from your background as an author, wherein you likely dealt with handling editors. Some of those encounters may have been more positive than others. What made them positive? (And similarly for the negative—what made those unpleasant?) Are there any takeaways? Try to do this as objectively as possible. For example, when thinking back to your past interactions with editors, try not to scapegoat or otherwise unfairly blame editors or “the journal” for negative outcomes (also, remember that one bad experience doesn’t define everything). In looking back, also try to calibrate your view of your past experiences and interactions with editors by taking into account an appreciation you may have now (versus years ago) of the time constraints faced by all of us, including editors. From an author’s perspective, the current manuscript/study may feel ultra-high priority (particularly at earlier stages in one’s career), while from the editor’s perspective it is simply impossible for each manuscript to be “highest” priority; try to balance these perspectives when serving as a handling editor.

Looking forward, utilize what you learn as an editor—you are gaining brand new knowledge, ahead of the pack. Serving as an editor will expand and enrich your perspectives on the science that you read; how fulfilling and important that is to you, personally, is another factor to weigh in deciding whether or not to commit to the editorial undertaking. While it can cause potential downstream conflicts, serving as an editor is generally an enviable position to be in, offering you a broader view of the field than you would have otherwise had; also, it can benefit your own writing practices and how you go about future interactions with editors. In short, you are in a highly privileged position by being an editor; you can leverage that, with appreciation and respect for all that it includes and entails. Basically, this is the “with great power comes great responsibility” adage from Spider-Man.

## Rule 3: Be fair and objective in judging the work, and treat all parties respectfully at all times

Remember that even distinguished scientists write bad papers (but they often know how to influence) and, conversely, little-known scientists write good papers. Don’t fall prey to name-recognition (of the authors, the institution, etc.) as a proxy for quality of the work, and give everyone a fair trial. This is essentially the idiom “don’t judge a book by its cover.” A related theme that suffuses this entire TSR is that you should treat all those involved—the authors, the reviewers, and the journal—with the utmost respect, even when (*especially when*) there may be differences of opinion.

## Rule 4: Don’t communicate about a manuscript outside of the journal system

This Rule is simple and clear enough, and should be abided by with the utmost stringency. If ever in doubt, first consult with a “higher-up” editor within the journal system (not outside it) for advice. Here, we mean “higher-up” as regards the handling hierarchy implicit in most editorial boards; for example, *PLOS Computational Biology* has 2 Editors-in-Chief (EIC), a deputy EIC, various “Section Editors” and “Academic Editors” (the latter is the pool of handling editors), and so on. The rationale for this Rule is multifold and stems from the reality that, sooner or later, things will go wrong; in such cases, “going rogue” with respect to the stricture of strict confidentiality makes a bad situation only worse. For example, you may get extensive pushback from authors on occasion, and in such cases it is crucial to have a single clean, explicit digital trail of all that has transpired with the manuscript under consideration. Such correspondence can occur via the internal tools and communication channels that are generally available at journals (Rule 8 mentions “journal management systems”). Also important, the reputation of the journal (and you as editor) depends on the equitableness of the review process, and there is no better way to ensure that than by adhering to these principles consistently and uniformly (i.e., for every submission).

## Rule 5: Remember the 90:10 rule: 10% of manuscripts will take 90% of your time

This principle holds in many areas of life, professional and otherwise. At some point in your editing activities, you will encounter a particular manuscript that consumes a disproportionate amount of time and effort. When that occurs, pause and try to determine *why* that is (before frustration potentially sets in). Next, it probably would be simplest and most beneficial (to all) for you to act swiftly—avoid problems early, when possible. For example, if the manuscript is at the very edge of the journal’s scope, and it is of borderline quality, then you can feel justified in the view that it is unlikely to be a good fit for the journal. One reason for this is that you risk having only poor reviews to go on, as the reviewers that you ultimately end-up assigning will be hard to come by, and they may not have detailed technical expertise that is germane to the specific questions/problems addressed in the work under consideration (given its incongruity with the journal). You can graciously reject such work sooner rather than later, on the grounds that it is legitimately out-of-scope for the journal. Always consider that a protracted review process benefits no one—neither you, the journal, nor the authors.

## Rule 6: Be prepared to adjudicate the majority of papers

For example, 3 reviews are most often split, frequently as a mix of “major” and “minor” revisions (and sometimes even “accept” or “reject”). While a clear consensus is reassuring, insisting on full unanimity among reviewers is unnecessary in proceeding to a decision. Furthermore, a fair-as-possible decision-making process often requires finesse, and may have to be quite nuanced—e.g., you may not reach a decision via a simple “majority vote,” as it is fine to up-weight or down-weight critiques based upon their quality. In other words, don’t be afraid to intervene in the overall process if you sense that doing so would improve the quality (of benefit to everyone) and/or the expediency or efficiency (of benefit to the authors and to the journal) of the overall process. Your intervention can be viewed as part of the review process, two key goals of which are assessing suitability and quality: (i) the suitability of the research for the journal and its audience; and (ii) the quality of the work itself. In assessing the work’s quality, there may be a need to call upon additional reviewers, and that’s fine; here, just remember that you’ll need to balance two countervailing forces: (i) obtaining an adequate number of sufficiently high-quality reviews (at least two, ideally more); and (ii) excessively lengthening the overall time-to-decision (the mean timescale can actually negatively or positively impact a journal’s reputation; it needs to be a balance). As another example of an “intervention,” as an editor you can exercise your judgment to draw the authors’ attention to any particular portions of a reviewer report that you feel merit greater attention than do other parts; this type of guidance from you can be especially helpful in cases of lengthy, sprawling reviews that offer a collection of valuable critiques as well as less-valuable critiques. At the end of the day, bear in mind that a journal’s reputation is tied to the quality of its decisions and its decision-making processes; as a handling editor, you are an integral component of those processes!

## Rule 7: View yourself as a matchmaker between manuscripts and the journal

In doing so, don’t lose sight of the journal scope and what they—the journal and the manuscript—are each trying to accomplish. As an extreme example, something like Watson and Crick’s DNA structure [3] and a review of RNA-binding proteins [4] are unlikely to appear in the same journal! (So-called “mega-journals,” a class of journal types pioneered by *PLOS ONE*, are an exception to this.) In general, *Journal ABC* and *Proceedings of XYZ* likely serve different purposes and target audiences. Manuscripts that are submitted to one journal versus another will ideally have some alignment with those respective goals, and it’s fairest to all (the authors, the journal, the community/audience) to heed that when serving as the work’s editor. In being mindful of context and scope, also remember to maintain perspective on whether the submitted work is a primary research article that reports new findings, versus a mostly methodological piece, a description of new software, a review article, a perspective piece, etc. Again, the criteria for evaluating those different *types* of publications differ, and it is vital to keep that in mind, particularly if you find yourself in a position of weighing multiple reviews that significantly diverge in their evaluation of the work (this becomes especially crucial if any peer-reviews appear to have lost sight of the article type and its scope/context).

## Rule 8: Use the reviews—and the journal’s tools—judiciously

Editors must draw heavily upon the expertise of reviewers, which typically number between 2 to 4. (As a handling editor, don’t be afraid to also solicit additional reviews, if need be!) Always bear in mind that reviewers are voluntarily contributing their time and expertise in performing the reviews, with the intent of helping you, as editor, reach a decision on whether or not the journal should publish a piece of work (ideally with a consensus among editors and all involved reviewers, though such does not have to be the case). Also, you will likely learn that finding reviewers for a manuscript can be a major time sink. The closer a potential reviewer is to the work being considered, the more likely you will have a win/win situation for all parties: (i) the more likely the individual is to accept the review assignment; and (ii) with such a reviewer, the manuscript will likely undergo a more careful, diligent review (see also Rule 5, regarding the benefits of a close overlap between the technical expertise of the reviewer and the subject/work reported in the manuscript).

As editor, helpful tools will generally be available to you via a “journal management system” (JMS). You can use these resources, which are automated to varying degrees, to help you identify conflicts of interest, detect plagiarism, assess the “fit” between a study and a specific journal (via a “Journal/Author Name Estimator [JANE]” tool [5]), etc. Indeed, there are now even AI-enabled utilities that will tell you what folks have published similar work, take a submission abstract and help you find potentially optimal reviewers, and so on (e.g., the “Artificial Intelligence Review Assistant” (AIRA) platform at the Frontiers journals [6]). Such tools can be critical if the scope of what you cover as an editor is broad. Finally, note that a JMS may well supply information on different individuals’ acceptance rates (their agreeing to review a manuscript), and possibly what they have reviewed; it behooves you to pay attention to that information, when available, as matching a manuscript with the best reviewers upfront saves you time.

## Rule 9: Beware conflicts of interest

**Fig 1:**
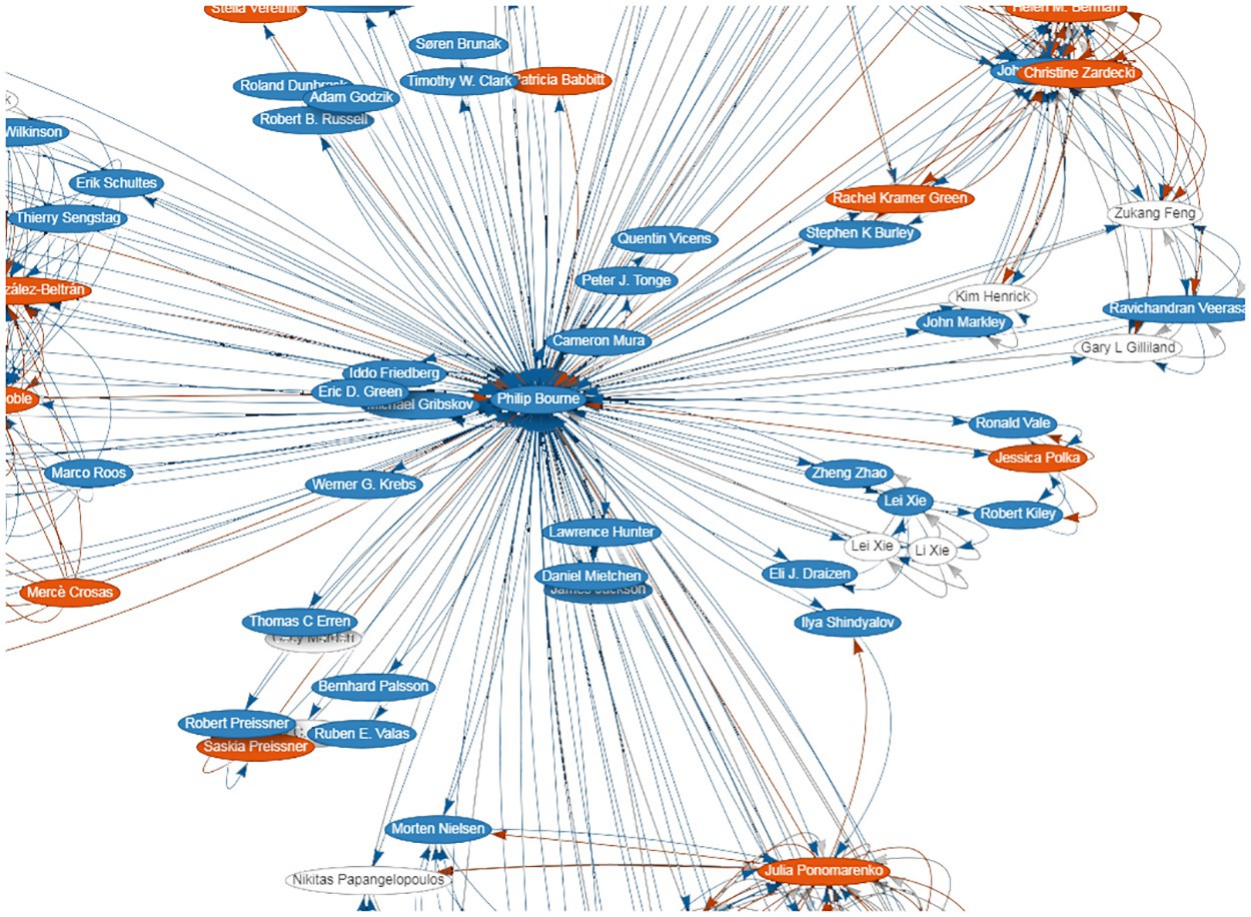
Co-authorship graphs offer direct, useful visual maps of professional linkages. As an example, here, we show a portion of Scholia’s auto-generated knowledge graph for Wikidata entity Q7183255 (https://www.wikidata.org/wiki/Q7183255), corresponding to researcher “Philip Bourne” and viewed via Scholia’s *Author* “aspect.” This graph was obtained directly from https://scholia.toolforge.org/author/Q7183255#coauthor-map. Being mindful of such linkages can help mitigate possible conflicts/entanglements among editors, reviewers, and authors.

Before agreeing to edit a particular submission, ask yourself how neutral and objective you are likely to be in this instance? This is phrased as “how” neutral because the realities of human behavior, emotions, and career trajectories are such that no one is 100% neutral. If for no other reasons, such may be the case simply because of the statistical properties of scientific social networks, stemming from the specialization and “influence propagation” in scientific subfields: the “six degrees of separation” phenomenon [7] may be more like “three degrees of influence” [8] in narrow scientific niches. This means, for example, that you’re statistically likely to be three hops away from having directly interacted with the folks whose work you were just invited to edit. To illustrate how easy a pitfall this is, consider Dr. Apple, who was a grad student with Prof. Bacon and is now finishing a postdoctoral position with Prof. Cook. Dr. Apple is about to start an independent position. Prof. Cook was a labmate with Prof. Bacon and Prof. Dunn in grad school 20 years ago. Even though there’s not a formal professional link between Dr. Apple and Prof. Dunn (and Cook and Dunn were “just” labmates back in the day), would it be as objective as possible for Dunn to handle a new Apple, Cook, et al. manuscript, versus someone else serving as its editor? This conflict will be apparent when you consider what the potential reviewer themselves has published and with whom. You can glean such information if you try to select reviewers based on their own publication record; in addition to ferreting out information via the JMS, perhaps you can also consider using coauthor knowledge graphs, easily accessible in Scholia [9], as a tool to facilitate this sort of preliminary “screening” (Fig 1). Finally, we note that the spirit of this Rule relates somewhat to Rule 4 above, regarding the privileged nature of the editing process and communications thereof. When in doubt, better to err on the side of neutrality and objectivity via non-involvement, versus potential entanglement or conflicts of interest.

## Rule 10: The review process should improve a paper in a finite amount of time

Here, we suggest being careful (mindful, disciplined) about how much time you allow the editing process to consume. As with many activities in life, a point of diminishing returns is reached sooner rather than later in the typical manuscript-review trajectory, and dedicating more time to the review process for a given paper won’t improve it (proportionately). Mostly, try to assess the paper, as guided by the peer-review process, try to facilitate its improvement (where possible), and remember to enjoy the process! In a small but definite way, as a handling editor you hold the future of science in your hand—use it wisely.
